# Prediction of Membrane Transport Proteins and Their Substrate Specificities Using Primary Sequence Information

**DOI:** 10.1371/journal.pone.0100278

**Published:** 2014-06-26

**Authors:** Nitish K. Mishra, Junil Chang, Patrick X. Zhao

**Affiliations:** Plant Biology Division, The Samuel Roberts Noble Foundation, Ardmore, Oklahoma, United States of America; University of Bern, Switzerland

## Abstract

**Background:**

Membrane transport proteins (transporters) move hydrophilic substrates across hydrophobic membranes and play vital roles in most cellular functions. Transporters represent a diverse group of proteins that differ in topology, energy coupling mechanism, and substrate specificity as well as sequence similarity. Among the functional annotations of transporters, information about their transporting substrates is especially important. The experimental identification and characterization of transporters is currently costly and time-consuming. The development of robust bioinformatics-based methods for the prediction of membrane transport proteins and their substrate specificities is therefore an important and urgent task.

**Results:**

Support vector machine (SVM)-based computational models, which comprehensively utilize integrative protein sequence features such as amino acid composition, dipeptide composition, physico-chemical composition, biochemical composition, and position-specific scoring matrices (PSSM), were developed to predict the substrate specificity of seven transporter classes: amino acid, anion, cation, electron, protein/mRNA, sugar, and other transporters. An additional model to differentiate transporters from non-transporters was also developed. Among the developed models, the biochemical composition and PSSM hybrid model outperformed other models and achieved an overall average prediction accuracy of 76.69% with a Mathews correlation coefficient (MCC) of 0.49 and a receiver operating characteristic area under the curve (AUC) of 0.833 on our main dataset. This model also achieved an overall average prediction accuracy of 78.88% and MCC of 0.41 on an independent dataset.

**Conclusions:**

Our analyses suggest that evolutionary information (i.e., the PSSM) and the AAIndex are key features for the substrate specificity prediction of transport proteins. In comparison, similarity-based methods such as BLAST, PSI-BLAST, and hidden Markov models do not provide accurate predictions for the substrate specificity of membrane transport proteins. *TrSSP: The Transporter Substrate Specificity Prediction Server*, a web server that implements the SVM models developed in this paper, is freely available at http://bioinfo.noble.org/TrSSP.

## Introduction

Membrane transport proteins, also known as transporters, transport hydrophilic substrates across hydrophobic membranes within an individual cell or between cells, and therefore play important roles in several cellular functions, including cell metabolism, ion homeostasis, signal transduction, binding with small molecules in extracellular space, the recognition process in the immune system, energy transduction, osmoregulation, and physiological and developmental processes [1]. Transporters represent a diverse group of proteins that differ in topology, energy coupling mechanism, and substrate specificity. In general, transport proteins are classified into channel/pore proteins, electrochemical transporters, active transporters, group translocators, and electron carriers. Transport proteins are primarily involved in the transportation of amino acids, cations, anions, sugars, proteins, mRNAs, electrons, water, and hormones. Transporters also transport various substrates [2], and multiple transporters may be associated with the transport of a particular substrate across cell membranes. To date, the classification of transporters based on different families/subfamilies as well as their specific substrates remains an important challenge in both structural and functional biology.

Early bioinformatics studies classified and assigned transport proteins to a particular transporter class based on multiple sequence alignment. Recently, several methods based on machine learning techniques have been developed [1], [3]–[6]. For example, Gromiha *et al*. [3] analyzed the amino acid composition of transport proteins and developed neural network-based models to classify these transport proteins as channel/pore proteins, electrochemical transporters, and active transporters. Ou *et al*. [7] further analyzed the amino acid composition and residue pair preferences of transport proteins and developed models to classify these proteins as channel/pore proteins, electrochemical transporters, and active transporters in six transporter classification families. Li *et al*. [5] developed a general machine learning based approach that integrated a set of rules, which were based on transporter sequence features learned from well-curated proteomes as guides, that covered major transporter families/subfamilies defined in the transporter classification database (TCDB, http://www.tcdb.org) [8].

One limitation of these methods, however, is that the prediction of substrate specificities of transporters using these general classification systems is difficult. Common protein sequence similarity search-based methods fail to predict the substrate specificities of transporters because very low similarity exists both within the same substrate transport protein classes and between different substrate transport protein classes. Recently, Schaadt *et al.* [9] analyzed the amino acid composition, pseudo-amino acid composition [10], pair amino acid composition [11], and multiple sequence alignment-based amino acid composition of *Arabidopsis thaliana* (*A. thaliana*) transport proteins and developed models to predict amino acid transporters, oligopeptides transporters, phosphate transporters, and hexose transporters [12]. These models defined protein sequences within the same transporter class as positive predictors and the protein sequences of other transporter classes as negative predictors. This method relies on the Euclidean distance between the amino acid composition of a given protein sequence and the mean composition of protein sequences of positive data for a particular substrate-specific class to calculate a score for each query sequence against each substrate-specific class. This score is then used to assign a substrate to the query sequence. More recently, Chen *et al.* [13] developed neural network-based models to predict substrate specificity for electron transporters, protein/mRNA transporters, ion transporters, and other transporters using a combination of amino acid composition, position-specific scoring matrices (PSSM), and biochemical properties such as the amino acid index (AAindex). Recently Barghash *et al*. [14] also developed a new method for classification of transporter proteins at transporter classification (TC) family level and substrate level (metal, phosphate, sugar and amino acid transporter) by using sequence similarity and sequence motif based methods. Their method works well for TC family classification but its performance is low for substrate level classification with F-scores around 40–75%.

In these previous studies, substrate-specific protein classes with insufficient data have been merged into one general class labeled “other transporters”. In this study, our main goal was to classify transport proteins into the maximum possible number of classes according to their transported substrates. To achieve this goal, we first constructed a substrate-specific transport protein dataset that consisted of seven classes of transporters exclusive to a particular substrate, i.e., amino acid transporters/oligopeptides, anion transporters, cation transporters, electron transporters, protein/mRNA transporters, sugar transporters, and other transporters. We also compiled a set of non-transporters as an extra class for background controls. For each substrate class, proteins of that class are considered as positive dataset while proteins of other classes are consider as negative dataset. We systemically analyzed the amino acid composition and physico-chemical composition of each protein and found compositional differences among different classes of proteins. We then developed support vector machine (SVM) models that utilized the different properties of transporter protein sequences. We found that our SVM model based on biochemical composition and evolutionary information (i.e., the PSSM profile) could accurately predict substrate specificity. We adopted a five-fold cross-validation evaluation schema to assess the performance of the developed models. Our best SVM models achieved accuracies of 84.08%, 69.19%, 76.59%, 81.43%, 77.96%, 78.57%, 66.73%, and 78.99% for amino acid transporters, anion transporters, cation transporters, electron transporters, protein/mRNA transporters, sugar transporters, other transporters, and non-transporters, respectively. We further evaluated the performance of these models on 180 independent proteins, and the best model achieved accuracies of 83.33%, 69.44%, 74.44%, 91.11%, 83.33%, 77.78%, 71.67%, and 80.00% for amino acid transporters, anion transporters, cation transporters, electron transporters, protein/mRNA transporters, sugar transporters, other transporters, and non-transporters, respectively. Finally, we developed *TrSSP: the Transporter Substrate Specificity Prediction Server*, which is a web server that implements and demonstrates these SVM models. The *TrSSP* web server is freely available at http://bioinfo.noble.org/TrSSP.

## Materials and Methods

### Data Compilation

We collected from the SwissProt UniProt database (release 2013_03) 10,780 transporter, carrier, and channel proteins that were well characterized at the protein level and had clear substrate annotations [15], [16]. We removed sequences that were fragmented. We also removed sequences annotated with more than two substrate specificities and biological function annotations that were based solely on sequence similarity. We manually curated the biological function annotations from the remaining sequences and compiled a total of 1,110 membrane transport protein sequences in which only one transporting substrate has been reported in the literature. We removed 210 sequences that showed greater than 70% similarity using CD-HIT software [17] (see **Figure S1** for details about the data compilation and curation processes). The 900 remaining transporter sequences were then divided into seven major classes of transporters based on their substrate specificity: 85 amino acid/oligopeptide transporters, 72 anion transporters, 296 cation transporters, 70 electron transporters, 85 protein/mRNA transporters, 72 sugar transporters, and 220 other transporters. We also compiled 660 non-transporters as an extra class of control proteins in our model development process by randomly sampling all the proteins in UniProt release 2013_03 excluding the 10,780 transporters.

We further divided the 1,560 compiled proteins into two datasets: 1) the main dataset, which consisted of 70 amino acid transporters, 60 anion transporters, 260 cation transporters, 60 electron transporters, 70 protein/mRNA transporters, 60 sugar transporters, 200 other transporters, and 600 non-transport proteins for a total of 1,380 proteins; and 2) an independent dataset, which consisted of 15 amino acid transporters, 12 anion transporters, 36 cation transporters, 10 electron transporters, 15 protein/mRNA transporters, 12 sugar transporters, 20 other transporters, and 60 non-transport proteins for a total of 180 proteins (see **Table S1** for a detailed dataset partition; all the sequences are available on our *TrSSP* web server at http://bioinfo.noble.org/TrSSP/). We applied a five-fold cross-validation schema on the 1,380 proteins in the main dataset to develop our SVM models. The performance of these SVM models was further tested and validated on the independent dataset of 180 proteins. To evaluate the prediction accuracy of the models for each class of proteins, proteins within the same class were considered a positive predictor and proteins from the remaining classes were considered a negative predictor.

### Extraction of multi-features from protein sequences for SVM model construction

#### Monopeptide composition

Amino acid composition is the best and most popular method to represent the features of a protein [18]. The monopeptide composition gives a fixed length pattern of 20 features. The amino acid composition of a protein is defined as the fraction of each amino acid within that protein. The percentage of each amino acid was calculated using the following formula:
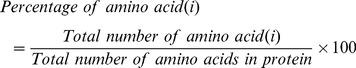
(1)where *i* represents one of the 20 standard amino acids.

#### Dipeptide composition

The dipeptide composition was used to encapsulate global information about each protein sequence. The dipeptide composition gives a fixed length pattern of 400 (20×20) features. Two consecutive amino acids are used to calculate the dipeptide composition information. This representation encompasses information about the amino acid composition as well as the local order of amino acids. The percentage of each dipeptide was calculated using the following formula:
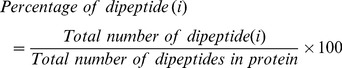
(2)where *i* can be any dipeptide of 400 possible dipeptides.

#### Physico-chemical composition

The physico-chemical composition is the composition of the physico-chemical class residues in each protein sequence. We calculated the percentage composition of charged (D, E, K, H, R), aliphatic (I, L, V), aromatic (F, H, W, Y), polar (D, E, R, K, Q, N), neutral (D, E, R, K, Q, N), hydrophobic (C, V, L, I, M, F, W), positively charged (H, K, R), negatively charged (D, E), tiny (A, C, D, G, S, T), small (E, H, I, L, K, M, N, P, Q, V), and large (F, R, W, Y) residues in each protein sequence [19]. We used the composition percentages of these 11 physico-chemical properties as an input feature to the SVM for model development [20].

#### Biochemical composition calculation

The biochemical composition of the amino acid residues was also used as an input feature to the SVM for model development. We used a set of 49 selected physical, chemical, energetic, and conformational properties to define the biochemical composition of each protein sequence [13]. These values are subsets of the AAIndex database [21], which has been successfully used to study protein folding and stability [22]–[24] and transporter classification [25]. We downloaded the 0–1 normalized values of these 49 properties from http://www.cbrc.jp/~gromiha/fold_rate/property.html; the details of each property are available at this website. We calculated the average of each biochemical property for each protein sequence using the following equation:
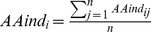
(3)


Where 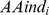 is the value for the *i*th biochemical property in a given protein sequence, 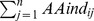 is the arithmetic sum of the *i*th biochemical property, and *n* is the length of the protein sequence. We therefore converted the biochemical properties of each protein sequence into a vector with a fixed size of 49.

### Position-specific scoring matrix (PSSM) profile

PSI-BLAST (Position-Specific Iterative Basic Local Alignment Search Tool) is a popular tool for the detection of distantly related proteins. PSI-BLAST calls BLAST (Basic Local Alignment Search Tool) to construct a profile or position-specific scoring matrix (PSSM) from the multiple alignments of the highest scoring hits in an initial BLAST search (default threshold e-value = 1e-3). The newly generated profile is then used iteratively to perform subsequent BLAST searches, and the result of each iteration is in turn used to refine the PSSM profile [26]. The PSSM therefore contains the probability of the occurrence of each type of amino acid residue at each position as well as insertions/deletions. Highly conserved positions receive high scores and weakly conserved positions receive near zero scores. We ran PSI-BLAST against the UniRef90 protein database (i.e., the non-redundant UniRef database with 90% sequence identity) [27] with the BLOSUM62 matrix [28]. We also used the SwissProt database [15] to generate the PSSM profile during our *TrSSP* web server development, which significantly reduced the computational runtime. The PSSM profile of a protein sequence extracted from PSI-BLAST was used to generate a 400-dimensional input vector to the SVM by summing all the rows in the PSSM that correspond to the same amino acid in the primary sequence. Every element in this input vector was then divided by the length of the sequence and scaled to the 0–1 range using the following standard linear function:
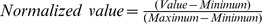
(4)where *Value* represents the individual final sum of the PSSM score for each amino acid [29].

### Cross-validation

Cross-validation is a practical and reliable way to test the predictive power of a newly developed model. The jack-knife or leave-one-out cross-validation (LOOCV) [30] and five-fold cross-validation are two commonly used techniques to evaluate a model. We used a five-fold cross-validation in the present SVM model development. In five-fold cross-validation, the dataset is partitioned into five equally sized random partitions [29], [31]. The methods of development and evaluation are conducted five times using four partitions as the training dataset and the remaining partition as the testing dataset. The performance of each model is computed as the average of the five runs.

### Support vector machines

The support vector machine (SVM) is a universal machine learning approximator based on the structural risk minimization (SRM) principle of statistical learning theory [32]. This technique is particularly attractive to biological sequence analysis due to its ability to handle noise and larger feature spaces [25]. We implemented SVM models using the SVM-Light software [33], which is freely available from http://svmlight.joachims.org/. SVM-Light enables the user to define the number of parameters and choose an inbuilt kernel, such as a linear, polynomial, sigmoid, or radial basis function (RBF) kernel. In this study, we tested linear, polynomial and RBF kernels for model development and found RBF performed better than other kernels. We also optimized both cost and gamma parameters (range of -j: 1- 4, -g: 1-e-5 - 10) of RBF kernel.

### Comparison to similarity search based methods

Sequence similarity remains the most popular method for the functional characterization of proteins. Therefore, we compared the performance of our SVM models for the prediction of substrate-specific transporter classes on both our main dataset and independent dataset to the following similarity search based methods: BLAST, PSI-BLAST, and hidden Markov models (HMM). In these similarity search based method development and evaluations, we used all unique transporter protein sequences without applying homology sequence filtering by using the CD-HIT tool.

#### BLAST

BLAST (Basic Local Alignment Search Tool) is one of the most popular bioinformatics tool for functional annotation of protein and nucleotide sequences [26], [34]. A BLAST search allows a user to search a query sequence against a library or database of sequences and find similar sequence in the library at a given cut-off threshold. The biological function of that hit sequence may be used to infer the function of the query sequence.

#### PSI-BLAST

PSI-BLAST is a tool that produces a PSSM constructed from a multiple alignment of the top-scoring BLAST hits to a given query sequence [26]. The position-specific matrix for round *n+1* is built from a constrained multiple alignment between the query sequence and the sequences found with a sufficiently low e-value in round *n*. This scoring matrix produces a profile designed to identify the key positions of conserved amino acids within a motif. Subtle relationships between proteins that are distant structural or functional homologs can often be detected when this profile is used to search a database; these relationships are often not detected by a BLAST search. Therefore, we used PSI-BLAST in addition to BLAST to detect remote homologies. We conducted an iterative search in which the sequences found in one round were used to build score models for the next round of searching. Three iterations of PSI-BLAST were conducted at different cutoff e-values. This module could predict any of the seven transporter and one non-transporter classes depending on the similarity of the query protein to the proteins in the dataset. If the top hit had an e-value lower than the cut-off threshold, then the annotation of the top hit was used as the predicted annotation of the query.

#### Hidden Markov models

HMMs are statistical models of the primary structure consensus of a sequence family. HMMs were initially developed for speech recognition [35]. In biological sequence analysis, HMMs are used to build a profile that captures important information about the degree of conservation at various positions in multiple alignments and the varying degree to which gaps and insertion are permitted. HMM-based methods, which work on a formal probabilistic basis, typically outperform methods based on pairwise comparison in both alignment accuracy and database search sensitivity and specificity. Further details about HMMs can be found in Krogh et al. [36]. We adopted HMM-based searching using a freely downloadable implementation of HMM, HMMER version 3.1b1 [37], which is freely available at http://hmmer.janelia.org.

To implement the HMM-based method, the entire dataset was divided into 5 subsets similar to the five-fold cross-validation schema [38]. Four subsets of sequences were multiply aligned using ClustalW2 [39], and alignment profiles were generated using ‘hmmbuild’ in HMMER 3.1.b1. This profile database was converted into compressed binary data files using ‘hmmpress’, and tested with the fifth subset of sequences using the ‘hmmscan’ module in HMMER 3.1b1.

### Assessment of prediction performances

Sensitivity, specificity, accuracy, coverage, and the Matthews correlation coefficient (MCC) were calculated for each test dataset in our five-fold cross validation to test the performance of each model. Parameters computed from each subset were averaged across all five subsets to obtain a final value.

Sensitivity was computed as 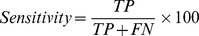
_,_ which evaluates the percentage of transporters that were correctly predicted as transporters.

Specificity was computed as 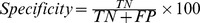
_,_ which evaluates the percentage of non-transporters that were correctly predicted as non-transport proteins.

Accuracy was computed as 
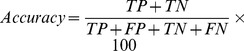
, which evaluates the overall percentage of transporters and non-transporters that were correctly predicted.

Coverage was computed as 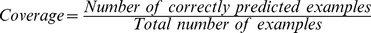, which provides a measure of the number of transporters that have been correctly predicted from the total dataset. This coverage is also commonly known as sensitivity or percentage of correct predictions/hits.

The Matthews correlation coefficient (MCC), which was computed as 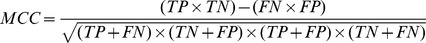
_,_ is a statistical parameter that assesses the quality of the binary classification for each model. The MCC accounts for both true and false positive predictions and is regarded as a balanced measure even when the two classes are different sizes. An MCC equal to 1 is regarded as a perfect prediction; an MCC close to 0 is regarded as a random prediction. In these formulas, TP (true positive) represents the number of correctly predicted transporters, TN (true negative) represents the number of correctly predicted non-transporters, FP (false positive) represents the number of non-transporters predicted as transporters, and FN (false negative) represents the number of transport proteins predicted as non-transporters.

All the parameters described above are threshold-dependent parameters; therefore, the performance of a model depends on a threshold. An analysis of the area under the curve (AUC) of the receiver operating characteristic (ROC) curve overcomes the threshold dependence of the above metrics. The ROC curve plots the true positive proportion (TP/TP+FN, i.e., sensitivity) against the false positive proportion (FP/FP+TN, i.e., 1 - specificity) for each model. The area under this ROC curve provides a single measure on which to evaluate the performance of each model. This well-known threshold-independent ROC analysis enables the evaluation of the performance of a binary classifier system as the discrimination threshold of that system is varied. An AUC of 1.0 indicates a perfect prediction and an AUC of 0.5 indicates that the prediction is no better than a random guess.

## Results

### Compositional biases

**Figure 1:**
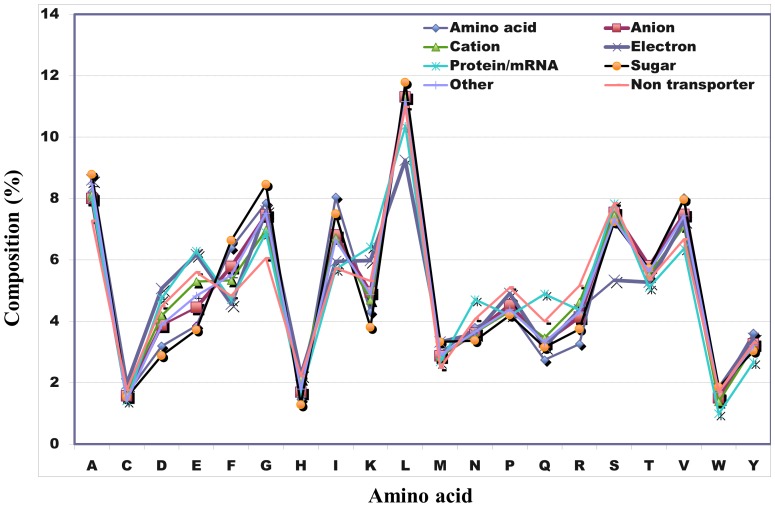
Amino acid composition of transporter proteins. The amino acid composition for each amino acid as a percentage of the total number of amino acids for amino acid transporters (blue diamonds), anion transporters (red squares), cation transporters (green triangles), electron transporters (purple x), protein/mRNA transporters (cyan asterisks), sugar transporters (orange circles), other transporters (plus signs), and non-transporters (orange dashes) are plotted.

**Figure 2:**
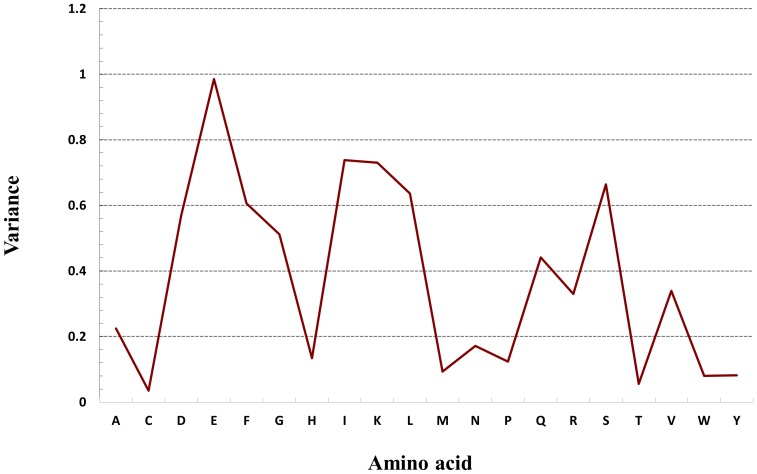
Variance inamino acid composition of transporter proteins. The variance in amino acid residues across amino acid transporters, anion transporters, cation transporters, electron transporters, protein/mRNA transporters, sugar transporters, other transporters, and non-transporters is plotted.

**Figure 3:**
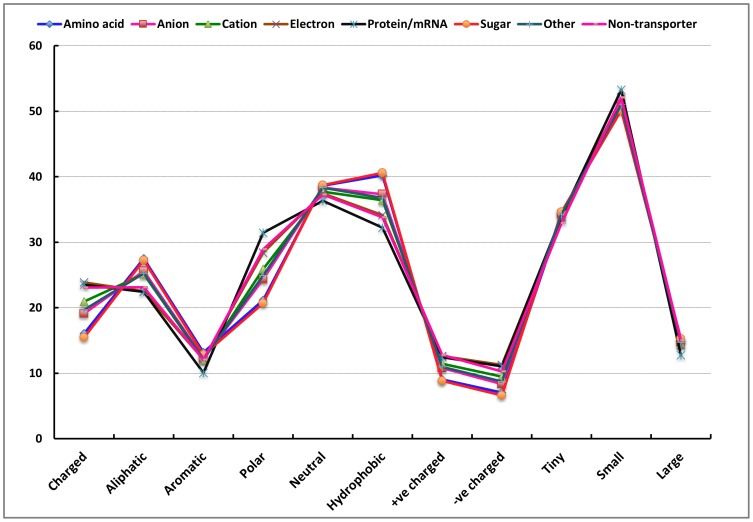
Physico-chemical composition of transporter proteins. The physico-chemical composition (as a percentage of the total amino acids) of amino acid transporters (blue diamonds), anion transporters (red squares), cation transporters (green triangles), electron transporters (purple x), protein/mRNA transporters (cyan asterisks), sugar transporters (orange circles), other transporters (plus signs), and non-transporters (orange dashes) are plotted for the following physico-chemical amino acid classes: charged, aliphatic, aromatic, polar, neutral, hydrophobic, positively charged, negatively charged, tiny, small, and large amino acids.

We computed the amino acid composition of eight classes of proteins, including seven substrate-specific transporter classes and one class of non-transporters. The composition of charged amino acids, such as Asp, Glu, Arg, and Lys as well as Gly, Ile, Phe, Gln, and Val (shown in **Figure 1**), differ among these eight classes. The variance in the amino acid concentrations among the eight classes is shown in **Figure 2**. The amino acids Asp, Glu, Lys, Phe, Gly, Ile, Leu, and Ser had a variance higher than 0.5. **Figure 3** shows the differences in the physico-chemical composition of the charged, polar, and hydrophobic amino acids among the eight classes. These variances suggest significant compositional differences among the different classes of substrate-specific transporter proteins.

### SVM performance on the main dataset

**Figure 4:**
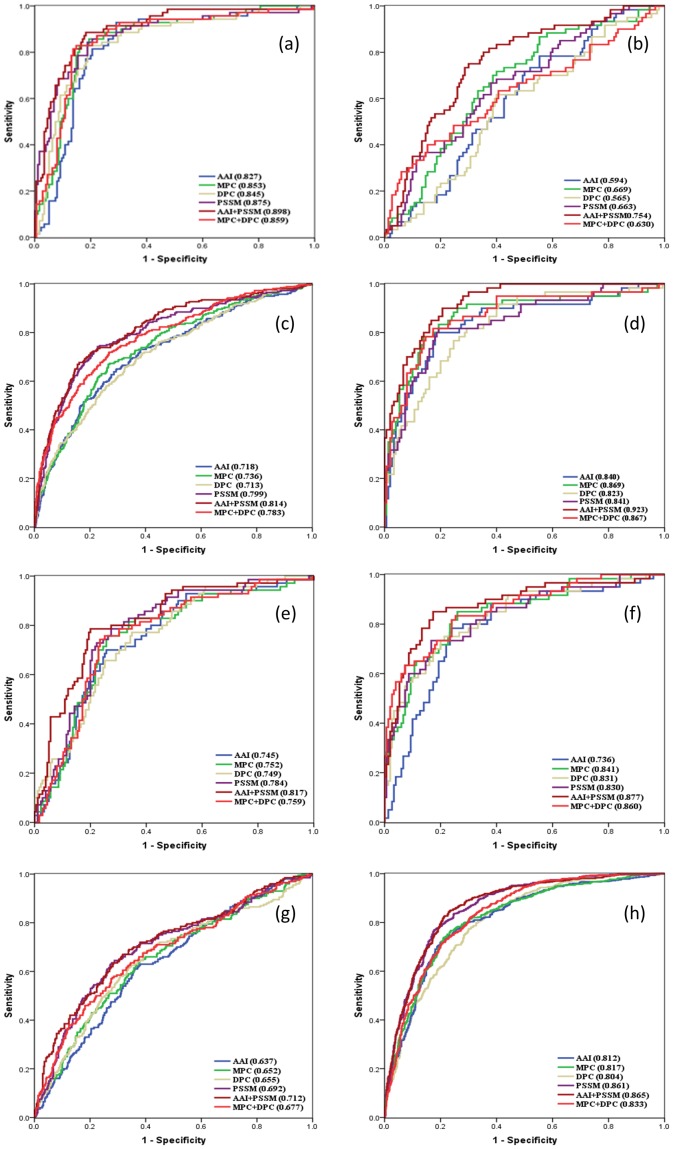
Receiver operating characteristic analysis. ROC analyses for (a) amino acid transporters; (b) anion transporters; (c) cation transporters; (d) electron transporters; (e) protein/mRNA transporters; (f) sugar transporters; (g) other transporters; and (h) non-transporters are plotted. Here AAI, MPC, DPC and PSSM denotes amino acid index, monopeptide composition, dipeptide composition and position specific scoring matrices respectively.

We used the amino acid composition, dipeptide composition, physico-chemical composition, biochemical composition (AAIndex), PSSM, and a combination of these properties to develop different models to discriminate amino acid/oligopeptides, anion, cation, electron, protein/mRNA, sugar, and other transporters and non-transporters. We then systematically evaluated the performance of each model using ROC analyses for (a) amino acid transporters; (b) anion transporters; (c) cation transporters; (d) electron transporters; (e) protein/mRNA transporters; (f) sugar transporters; (g) other transporters; and (h) non-transporters (see results in **Figure 4**).

**Table 1** shows the average sensitivity, specificity, accuracy, and MCC of all seven substrate-specific transporter classes using different SVM models. **Table 2** shows the average sensitivity, specificity, accuracy, and MCC of our best models for eight classes, which include the seven substrate-specific transporter classes and the non-transporter class. These results show that the AAIndex+PSSM-based model outperforms the other models. We also tested models that used a combination of PSSM and other compositions; however, the overall performance was not improved in these models. Our best model that integrates the biochemical composition (AAIndex) and the PSSM profile achieved an accuracy of 84.08%, 69.19%, 76.59%, 81.43%, 77.96%, 78.57%, 66.73%, and 78.99% for amino acid transporters, anion transporters, cation transporters, electron transporters, protein/mRNA transporters, sugar transporters, and other transporters, respectively. Similarly, this model achieved MCC values of 0.65, 0.32, 0.47, 0.60, 0.51, 0.52, 0.29, and 0.58 and AUC values of 0.898, 0.754, 0.814, 0.923, 0.817, 0.877, 0.712, and 0.865 for amino acid transporters, anion transporters, cation transporters, electron transporters, protein/mRNA transporters, sugar transporters, and other transporters, respectively.

**Table 1:** The average sensitivity, specificity, accuracy, and MCC for all seven substrate-specific transporter classes for different models.

Method	Sensitivity (%)	Specificity (%)	Accuracy (%)	MCC
AAC	74.65	73.22	73.74	0.46
DPC	71.36	71.17	71.32	0.40
PHC	70.63	70.42	70.60	0.38
AAI	71.54	71.98	71.80	0.40
PSSM	74.00	76.03	75.48	0.47
AAC+AAI	73.84	73.36	73.37	0.44
AAC+DPC	73.01	74.23	73.85	0.44
AAC+PHC	73.56	73.39	73.40	0.44
AAC+PSSM	72.33	74.62	73.87	0.44
DPC+AAI	70.28	72.09	71.67	0.40
DPC+PHC	72.15	73.13	72.90	0.43
DPC+PSSM	69.87	72.06	71.37	0.40
AAI+PHC	69.32	71.50	70.79	0.38
AAI+PSSM	76.19	77.17	76.69	0.49
AAC+DPC+AAI	73.01	74.32	73.89	0.44
AAC+DPC+PHC	73.04	75.36	74.75	0.46
AAC+DPC+PSSM	73.43	73.73	73.64	0.44
AAC+AAI+PHC	73.01	73.82	73.51	0.44
AAC+AAI+PSSM	72.34	74.62	73.90	0.44

AAC: Amino acid composition; DPC: Dipeptide composition; AAI: Biochemical composition (AAIndex); PHC: Physico-chemical class composition; PSSM: Position-specific scoring matrix.

**Table 2:** The performances of the best models on the main dataset for different substrate-specific transporter classes.

Transporter class	Sensitivity (%)	Specificity (%)	Accuracy (%)	MCC	AUC
Amino acid	85.72	83.43	84.08	0.65	0.898
Anion	71.67	69.00	69.19	0.32	0.754
Cation	70.00	79.29	76.59	0.47	0.814
Electron	85.00	80.00	81.43	0.60	0.923
Protein/mRNA	74.29	79.43	77.96	0.51	0.817
Sugar	76.67	79.33	78.57	0.52	0.877
Other	66.50	68.50	66.93	0.29	0.712
Non-transporter	79.67	78.46	78.99	0.58	0.865

Although SVM models using the PSSM profile, which was generated with UniRef90, performed well (**Table S2** and **Table S4**), the PSSM profile takes a long time to compute due to the size of the UniRef90 database. Therefore, we used the UniProtKB/SwissProt release 2013-03 as the reference dataset in order to reduce the PSSM computation time. We achieved a similar result when this PSSM profile was used in the SVM model, and the PSSM generation process was about 10 times faster than the generation process that used UniRef90. The hybrid model that included the biochemical composition and this PSSM profile achieved an accuracy of 83.27%, 67.14%, 76.15%, 81.43%, 74.69%, 78.57%, 66.71%, and 78.12% and an accuracy of 84.44%, 68.33%, 71.11%, 81.67%, 83.33%, 80.56%, 69.44%, and 80.00% for amino acid transporters, anion transporters, cation transporters, electron transporters, protein/mRNA transporters, sugar transporters, and other transporters, respectively, of which the performances were analyzed based on both the main dataset and the independent dataset (see **Table S3** and **Table S5**). Confusion matrix of training data suggests that our best model working well for each class of transporters in main dataset (**Table S6**).

### SVM performance on the independent dataset

We used an independent dataset of 15 amino acid transporters, 12 anion transporters, 36 cation transporters, 10 electron transporters, 15 protein/mRNA transporters, 12 sugar transporters, and 20 other transporters (see **Table S1**) to further evaluate the performance of our SVM models. The best SVM model, which used both the biochemical composition and the UniRef90-generated PSSM profile, achieved an accuracy of 83.33%, 69.44%, 74.44%, 91.11%, 83.33%, 77.78%, 71.67%, and 80.00% for amino acid transporters, anion transporters, cation transporters, electron transporters, protein/mRNA transporters, sugar transporters, and other transporters, respectively (see **Table 3** for details).

**Table 3:** The performances of the best models on the independent dataset for different substrate-specific transporter classes.

Transporter class	Sensitivity (%)	Specificity (%)	Accuracy (%)	MCC
Amino acid	93.33	82.42	83.33	0.49
Anion	75.00	69.05	69.44	0.23
Cation	75.00	74.31	74.44	0.41
Electron	80.00	91.78	91.11	0.50
Protein/mRNA	93.33	82.42	83.33	0.49
Sugar	91.67	76.79	77.78	0.38
Other	60.00	73.13	71.67	0.23
Non-transporter	76.67	81.67	80.00	0.57

We also tested the hybrid model that used the biochemical composition and the UniProt/SwissProt-based PSSM profile on the independent dataset. This model achieved an accuracy of 84.44%, 68.33%, 71.11%, 81.67%, 83.33%, 80.56%, 69.44%, and 80.00% for amino acid transporters, anion transporters, cation transporters, electron transporters, protein/mRNA transporters, sugar transporters, and other transporters, respectively. Confusion matrix of independent data suggests that our best model working well for independent data of each class of transporters (**Table S7**).

### Comparisons with other classification models

#### Substrate specificity classification

Chen *et al*. [13] developed models for four substrate-specific transporter classes: electron transporters, protein/mRNA transporters, ion transporters, and other transporters. The use of only four transporter classes makes difficult a direct comparison to our models, which were developed on seven transporter classes. We therefore predicted substrate specificity using their *TTRBF* web server at http://rbf.bioinfo.tw/~sachen/TTpredict/Transporter-RBF.php, and grouped our cation and anion transporters into the ion transporter class and our amino acid transporters, sugar transporters, and other transporters into the other transporter class for our independent dataset We used a threshold of 0.65 to differentiate between non-transporters (below 0.65) and transporters (greater than or equal to 0.65). **Table 4** provides details about the comparison between our SVM models and the models from the Chen et al. *TTRBF* web server. Our models outperformed the Chen et al. models in all cases except in the case of ion transporters by an extremely small margin (**Table 5**). Furthermore, our models have an average coverage of 83.21% compared to an average coverage of 65.33% using the Chen et al. models.

**Table 4:** A comparison of the performance of our model with TTRBF.

Transporter class	Total	Correct predictions by our models	Correct predictions by TTRBF	*Transporter Class
Amino acid^%^	15	14	5	Other
Anion^∧^	12	9	5	Ion
Cation^∧^	36	27	31	
Electron	10	8	6	Electron
Protein/mRNA	15	14	13	Protein/mRNA
Sugar^%^	12	11	11	Other
Other^%^	20	12	3	Other
Non-transporter^$^	60	46	11	

* Four substrate-specific transporter classes described in Chen et al. [13]; $ Non-transporter, % other class; ∧ ion transporter.

**Table 5:** A comparison of the coverage metric of our models with TTRBF.

Transporter class	Total	Coverage (%) of our models	Coverage (%) of TTRBF
Electron	10	80.00	60.00
Ion	48	75.00	75.00
Protein/mRNA	15	93.33	86.67
Other	47	76.60	40.43
**Average**		**83.21**	**65.53**

### Transporter and non-transporter classification

We also compared the performance of transporter and non-transporter classification between our model and the model of Ou *et al* [7] using their web server at http://rbf.bioinfo.tw/~sachen/TCpredict/Transporter-RBF.php. The Ou *et al.* model predicted transporters and non-transporters with an accuracy of 78.89% and MCC of 0.53, whereas our models predicted transporters and non-transporters with an accuracy of 80.00% and MCC of 0.57. This result therefore suggests that our models outperform the Ou *et al*. model for transporter and non-transporter classification.

### BLAST performance

Sequence similarity remains the most popular method for the functional characterization of proteins. In general, if the performance of BLAST-based methods is acceptable, then the development of new models is unnecessary. In our study, we used BLAST to discriminate between the seven substrate-specific transporter classes and the non-transporter class. We used the coverage metric to evaluate the performance of the BLAST method on the prediction of substrate specificity in our five-fold cross-validation. For the main dataset, the BLAST results achieved a coverage range between 25.00% and 75.71% (see **Table 6**). Similarly, for the independent dataset, the BLAST results at an e-value of 1e-4 achieved a coverage range between 0.00% and 41.67% at an e-value of 1e-4 (see **Table 7**). These results suggest that BLAST almost failed to discriminate transporters. The BLAST method performed poorly for the prediction of anion transporters, electron transporters, and protein/mRNA transporters on the main dataset and several classes of transporters on the independent dataset. The performance of the BLAST method decreased further when we applied more stringent e-value cutoff thresholds.

**Table 6:** The performance (coverage metric) of the BLAST search on the main dataset using the standard five-fold cross-validation.

Transporter class	Coverage (%) at E-value cutoff thresholds		
	1E-4	1E-10	1E-50
Amino acid	75.71	75.71	52.86
Anion	50.00	48.33	36.67
Cation	66.54	60.38	40.77
Electron	25.00	20.00	11.67
Protein/mRNA	32.86	31.43	20.00
Sugar	68.33	65.00	50.00
Other	58.00	57.00	37.00
Non-transporter	32.17	24.67	7.50

**Table 7:** The performance (coverage metric) of the BLAST search on the independent dataset.

Transporter class	Coverage (%) at E-value cutoff thresholds		
	1E-4	1E-10	1E-50
Amino acid	33.33	13.33	0.00
Anion	25.00	16.67	0.00
Cation	36.11	36.11	16.67
Electron	0.00	0.00	0.00
Protein/mRNA	0.00	0.00	0.00
Sugar	41.67	41.67	16.67
Other	10.00	10.00	0.00
Non-transporter	10.00	8.33	3.00

### PSI-BLAST performance

In this study, we used PSI-BLAST in addition to BLAST because PSI-BLAST has the added capability of detecting remote homologies. For the main dataset, the PSI-BLAST search results achieved a coverage range between 30.00% and 74.29% at an e-value of 1e-4 (see **Table 8**). Similarly, for the independent dataset, the PSI-BLAST search results achieved a coverage range between 0.00% and 41.67% at an e-value of 1e-4 (see **Table 9**). Therefore, PSI-BLAST also failed to discriminate between transport and non-transport proteins. The PSI-BLAST method performed poorly for the prediction of anion transporters, electron transporters, and protein/mRNA transporters on the main dataset, and failed to predict several transporter classes on the independent dataset. The performance of the PSI-BLAST method decreased further when we applied more stringent e-values. The BLAST and PSI-BLAST results therefore suggest that similarity-based methods are not suitable for the prediction of substrate-specific transporter classes.

**Table 8:** The performance (coverage metric) of the PSI-BLAST search on the main dataset using a five-fold cross-validation.

Transporter class	Coverage (%) at E-value cutoff thresholds		
	1E-4	1E-10	1E-50
Amino acid	74.29	74.29	52.86
Anion	40.00	38.33	31.67
Cation	68.46	62.69	41.54
Electron	30.00	30.00	13.33
Protein/mRNA	34.29	32.86	21.43
Sugar	68.33	65.00	51.67
Other	59.00	56.50	38.50
Non-transporter	34.67	25.50	7.67

**Table 9:** The performance (coverage metric) of the PSI-BLAST search on the independent dataset.

Transporter class	Coverage (%) at E-value cutoff thresholds		
	1E-4	1E-10	1E-50
Amino acid	26.67	13.33	0.00
Anion	33.33	16.67	0.00
Cation	36.11	36.11	19.44
Electron	0.00	0.00	0.00
Protein/mRNA	0.00	0.00	0.00
Sugar	41.67	41.67	16.67
Other	10.00	10.00	0.00
Non-transporter	10.00	8.33	3.33

### HMM performance

We used HMM profiles that were built using the ClustalW2 multiple sequence alignment software in the HMMER 3.1b1 software package to search for similar sequences. As shown in **Table 10** **,** for the main dataset, the HMM results had a coverage range between 3.33% and 47.14% at an e-value of 1e-4. Similarly, for the independent dataset, the HMM results achieved a coverage range between 0.0% and 41.66% at an e-value of 1e-4 (**Table 11** **)**. This analysis suggests that the HMM-based profile searching method performs poorly for the prediction of substrate-specific transporter classes, and completely failed for a few classes.

**Table 10:** The performance (coverage metric) of the HMM search on the main dataset using a five-fold cross-validation.

Transporter class	Coverage (%) at E-value cutoff thresholds		
	1E-4	1E-10	1E-50
Amino acid	47.14	42.86	11.43
Anion	18.33	15.00	8.33
Cation	8.46	6.54	4.23
Electron	3.33	1.67	0.00
Protein/mRNA	5.71	2.86	0.00
Sugar	46.67	46.67	40.00
Other	20.00	17.00	9.00
Non-transporter	0.00	0.00	0.00

**Table 11:** The performance (coverage metric) of the HMM search on the independent dataset.

Transporter class	Coverage (%) at E-value cutoff thresholds		
	1E-4	1E-10	1E-50
Amino acid	40.00	40.00	20.00
Anion	41.66	41.66	8.33
Cation	11.11	8.33	5.55
Electron	10.00	10.00	0.00
Protein/mRNA	0.00	0.00	0.00
Sugar	25.00	25.00	25.00
Other	10.00	5.00	0.00
Non transporter	0.00	0.00	0.00

### Proteome-scale transporter annotation

We applied our best model to predict transporters at the proteome level for Human, Drosophila, Yeast, *Escherichia coli (E. coli)*, and *A. thaliana* proteins. To perform a proteome-level transporter analysis, we collected experimentally annotated full-length protein sequences from SwissProt release 2013-06. The details of this analysis are summarized in **Table 12**; the entire prediction for each organism is available on the *TrSSP* web server (http://bioinfo.noble.org/TrSSP/?dowhat=Datasets). Our results suggest that *E. coli* has the largest percentage of transporter proteins followed by *A. thaliana*; humans have the lowest percentage of transporter proteins. We also observed that amino acid and sugar transporters represent the smallest percentage of transporters in all organisms tested except *E. coli*, and cation and electron transporters represent the highest percentage of transporters in all organisms tested. A complete list of sequences and their substrate specificities are available on the *TrSSP* web server.

**Table 12:** The number of predicted transporters in Human, Drosophila, *E. coli*, Yeast and *A. thaliana*.

	Human	Drosophila	E. coli	Yeast	A. thaliana
Total number of/Percentage of protein sequences	20,254	3,198	22,142	7,794	12,197
Total transporters	2,112	448	5,939	960	2,440
	(10.43%)	(14.01%)	(26.82%)	(12.32%)	(20.01%)
Amino acid transporters	216	25	705	170	369
	(1.07%)	(0.78%)	(3.18%)	(2.18%)	(3.02%)
Anion transporters	661	70	492	251	776
	(3.26%)	(2.19%)	(2.13%)	(3.22%)	(6.36%)
Cation transporters	1,001	204	2,575	329	1084
	(4.94%)	(6.38%)	(11.63%)	(4.22%)	(8.89%)
Electron transporters	600	153	3081	329	547
	(2.96%)	(4.78%)	(13.91%)	(4.22%)	(4.48%)
Protein transporters	913	223	1,644	376	659
	(4.51%)	(6.97%)	(7.42%)	(4.82%)	(5.40%)
Sugar transporters	402	51	1,041	173	492
	(1.98%)	(1.59%)	(4.70%)	(2.22%)	(4.03%)
Other transporters	623	96	1,087	274	670
	(3.08%)	(3.00%)	(4.91%)	(3.52%)	(5.49%)

## Discussion

The experimental characterization of transporters at the substrate-specific level is difficult and time consuming. Substrate-specific transporter characterization is also difficult in bioinformatics studies because transporters have remote homologies with other proteins both within and between protein classes. Advanced computational techniques that identify substrate-specific transport proteins from their primary sequences are urgently needed.

Although Schaadt *et al*. [9] have previously developed models to predict the substrate specificity of transporters for *A. thaliana* proteins, one limitation of their models is that only 61 proteins were used in the training dataset for model development. These models were also not made available through software or a web server for users to analyze their own sequences. Chen *et al*. [13] have developed models to predict the substrate specificity for electron transporters, protein/mRNA transporters, ion transporters, and other transporters, and more recently improved this method to differentiate transporters from non-transporters using a probability distribution function for each query protein. This improved method, which is essentially a combination of the original Chen *et al.* model and the Ou *et al.* model, is limited in that the proposed threshold of 0.65 is not reliable for the prediction of transporters. Barghash *et al*. [14] model is also limited to classifying transporters of only four substrates and at TC family/subfamily level.

The models developed in the present study can simultaneously predict whether a query protein is a transporter or non-transporter protein and its substrate specificity for seven transporter protein classes. One advantage of our model is that it can differentiate cation and anion transporters. Our PSSM-based model demonstrated superior performance with respect to substrate specificity prediction. However, this model was computationally demanding when the PSSM profile was generated from the UniRef90 database. We observed that our *TrSSP* web server would take approximately 6–15 minutes per sequence to run when the UniRef90 database was used for PSSM generation. To significantly reduce the PSSM computational time, we implemented parallel computing for PSSM generation and used the UniProt/SwissProt database as the reference database, which reduced the runtime of our *TrSSP* server to approximately 10 minutes for approximately 200 sequences with no impact on model performance.

## Conclusions

We observed that sequence-similarity based methods such as BLAST, PSI-BLAST, and HMM were unable to accurately predict substrate-specific transporter classes. These results were expected because transporter proteins are diverse and have a remote homology both within and between transporter classes. Our current study suggests that we can predict the substrate specificity of transport proteins using SVM models that incorporate the biochemical composition, amino acid composition, and PSSM profile of transporter proteins. Our five-fold cross-validation method on the main dataset revealed that the best model, which included the AAIndex and the PSSM profile, achieved a prediction accuracy of 84.08%, 69.19%, 76.59%, 81.43%, 77.96%, 78.57%, 66.73%, and 78.99% for amino acid transporters, anion transporters, cation transporters, electron transporters, protein/mRNA transporters, sugar transporters, and other transporters, respectively. This model also achieved similar prediction accuracy on the independent dataset. Therefore, the models developed in the present study not only outperform the current available classifiers but also predict substrate specificity for more transporter classes than previous methods.

### Web server

We have developed a web server based on this work, which is freely available at http://bioinfo.noble.org/TrSSP. Users can upload or paste protein sequences in Fasta format for transporter and substrate prediction. We have provided six prediction modules on this web server: an amino acid composition based SVM, an AAIndex based SVM, a PSSM (SwissProt) based SVM, an AAIndex/PSSM (SwissProt) hybrid SVM, a PSSM (UniRef90) based SVM, and an AAIndex/PSSM (UniRef90) hybrid SVM. The *TrSSP* web server uses the amino acid composition module as the default. For the amino acid composition and AAIndex based modules, users can upload/paste a maximum of 2,000 sequences for batch predictions. Due to the high computational demand, we provide 1) PSSM (SwissProt) or AAIndex/PSSM (SwissProt) hybrid modules where users can upload/paste a maximum of 1,000 sequences and 2) PSSM (UniRef90) or AAIndex/PSSM (UniRef90) hybrid modules where users can upload/paste a maximum of 280 sequences for batch predictions. Although we have implemented a parallel PSSM generation, the PSSM-based modules have a long runtime; therefore, we provide users with the option to enter their email address to retrieve their prediction at a later time (within 120 days).

## Supporting Information

Figure S1 **Data compilation and curation.** A flowchart of the data compilation and curation processes. The values to the left of the text indicate the number of proteins that were available to the analysis at each step.
(TIF)

Table S1 The numbers of samples in the main dataset and independent dataset for different transporter classes.
(DOCX)

Table S2 The performances of the SwissProt-based PSSM models on the main dataset.
(DOCX)

Table S3 The performances of the hybrid AAIndex and SwissProt-based PSSM models on the main dataset.
(DOCX)

Table S4 The performances of the SwissProt-based PSSM models on the independent dataset.
(DOCX)

Table S5 The performances of the hybrid AAIndex and SwissProt-based PSSM models on the independent dataset.
(DOCX)

Table S6 Confusion matrix of best SVM models (AAindex+PSSM) on main dataset.
(DOCX)

Table S7 Confusion matrix of best SVM models (AAindex+PSSM) on independent dataset.
(DOCX)
