# Supplementary figures and images for: Prediction of Membrane Transport Proteins and Their Substrate Specificities Using Primary Sequence Information
Source: PLoS One. 2014 Jun 26;9(6):e100278. doi: 10.1371/journal.pone.0100278 (PMC4072671; doi:10.1371/journal.pone.0100278)

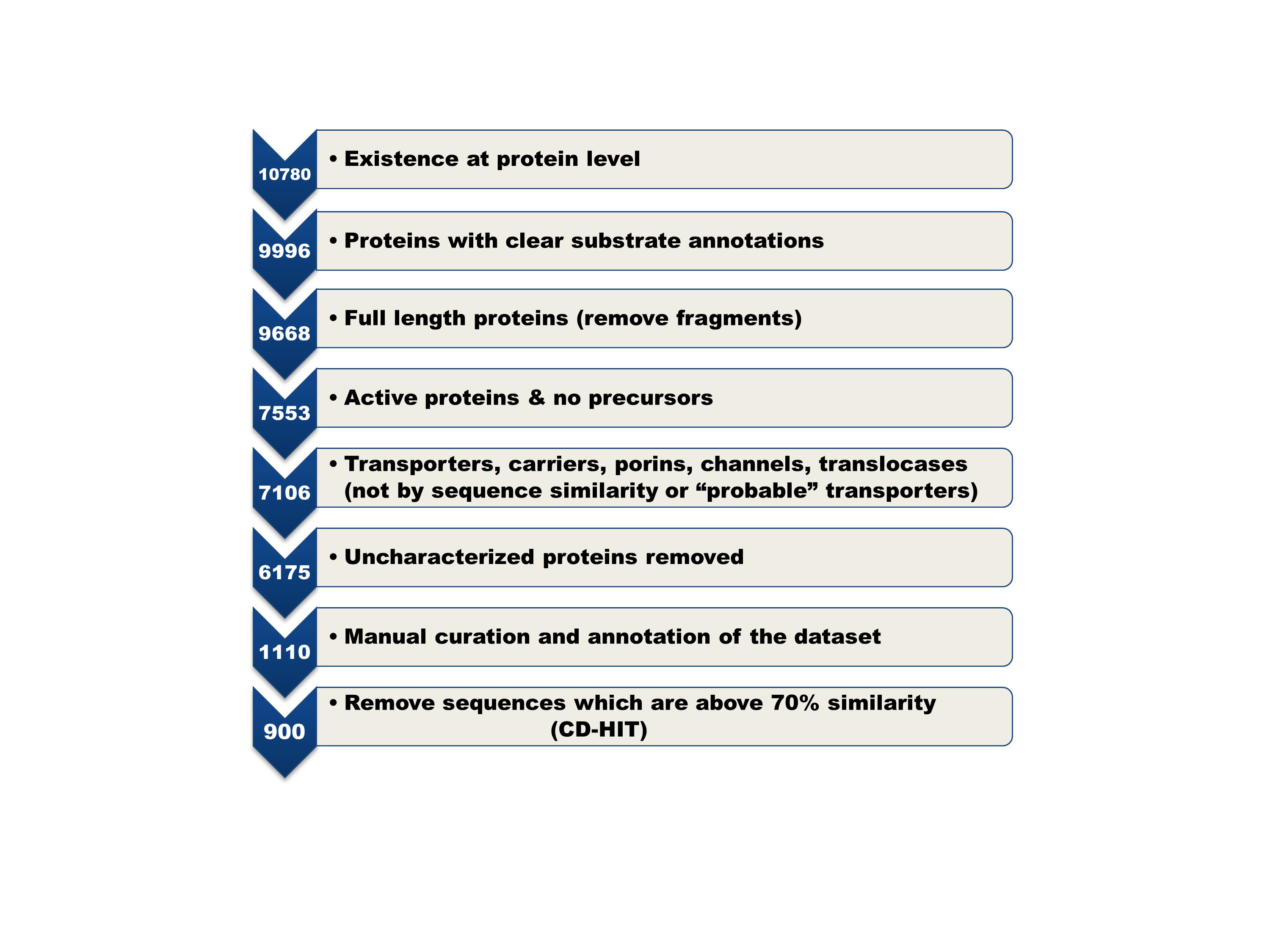

Supplement: Figure S1 — Data compilation and curation. A flowchart of the data compilation and curation processes. The values to the left of the text indicate the number of proteins that were available to the analysis at each step. (TIF) [file pone.0100278.s001.tif]
