# Supplementary material for: Prediction of Membrane Transport Proteins and Their Substrate Specificities Using Primary Sequence Information
Source: PLoS One. 2014 Jun 26;9(6):e100278. doi: 10.1371/journal.pone.0100278 (PMC4072671; doi:10.1371/journal.pone.0100278)
Supplement: Table S1 — The numbers of samples in the main dataset and independent dataset for different transporter classes. (DOCX) [file pone.0100278.s002.docx]

**Table S1**. The numbers of samples in the main dataset and independent dataset for different transporter classes.

| **Transporter class** | **Samples in the main dataset** | **Samples in the independent dataset** |
| --- | --- | --- |
| Amino acid | 70 | 15 |
| Anion | 60 | 12 |
| Cation | 260 | 36 |
| Electron | 60 | 10 |
| Protein/mRNA | 70 | 15 |
| Sugar | 60 | 12 |
| Other | 200 | 20 |
| ***Total transporters*** | ***780*** | ***120*** |
| Non-transporters | 600 | 60 |
| ***Total proteins*** | ***1380*** | ***180*** |
