# Supplementary material for: Prediction of Membrane Transport Proteins and Their Substrate Specificities Using Primary Sequence Information
Source: PLoS One. 2014 Jun 26;9(6):e100278. doi: 10.1371/journal.pone.0100278 (PMC4072671; doi:10.1371/journal.pone.0100278)
Supplement: Table S2 — The performances of the SwissProt-based PSSM models on the main dataset. (DOCX) [file pone.0100278.s003.docx]

**Table S2**. The performances of the SwissProt-based PSSM models on the main dataset.

| **Transporter class** | **Sensitivity** | **Specificity** | **Accuracy** | **MCC** |
| --- | --- | --- | --- | --- |
| Amino acid | 82.86 | 83.43 | 83.27 | 0.62 |
| Anion | 65.00 | 67.33 | 66.67 | 0.30 |
| Cation | 75.00 | 73.38 | 73.85 | 0.45 |
| Electron | 85.00 | 80.00 | 81.43 | 0.58 |
| Protein/mRNA | 71.43 | 76.00 | 74.69 | 0.44 |
| Sugar | 73.33 | 79.33 | 77.62 | 0.50 |
| Other | 72.50 | 63.80 | 66.29 | 0.33 |
| Non-transporter | 83.33 | 74.10 | 78.12 | 0.57 |
