# Supplementary material for: Prediction of Membrane Transport Proteins and Their Substrate Specificities Using Primary Sequence Information
Source: PLoS One. 2014 Jun 26;9(6):e100278. doi: 10.1371/journal.pone.0100278 (PMC4072671; doi:10.1371/journal.pone.0100278)
Supplement: Table S3 — The performances of the hybrid AAIndex and SwissProt-based PSSM models on the main dataset. (DOCX) [file pone.0100278.s004.docx]

**Table S3**. The performances of the hybrid AAIndex and SwissProt-based PSSM models on the main dataset.

| **Transporter class** | **Sensitivity** | **Specificity** | **Accuracy** | **MCC** |
| --- | --- | --- | --- | --- |
| Amino acid | 80.00 | 84.57 | 83.27 | 0.62 |
| Anion | 73.33 | 64.67 | 67.14 | 0.34 |
| Cation | 72.31 | 77.69 | 76.15 | 0.47 |
| Electron | 80.00 | 82.00 | 81.43 | 0.58 |
| Protein/mRNA | 72.86 | 75.43 | 74.69 | 0.45 |
| Sugar | 80.00 | 78.00 | 78.57 | 0.51 |
| Other | 69.00 | 65.80 | 66.71 | 0.33 |
| Non-transporter | 81.50 | 75.51 | 78.12 | 0.57 |
