# Supplementary material for: Prediction of Membrane Transport Proteins and Their Substrate Specificities Using Primary Sequence Information
Source: PLoS One. 2014 Jun 26;9(6):e100278. doi: 10.1371/journal.pone.0100278 (PMC4072671; doi:10.1371/journal.pone.0100278)
Supplement: Table S4 — The performances of the SwissProt-based PSSM models on the independent dataset. (DOCX) [file pone.0100278.s005.docx]

**Table S4**. The performances of the SwissProt-based PSSM models on the independent dataset.

| **Transporter class** | **Sensitivity** | **Specificity** | **Accuracy** | **MCC** |
| --- | --- | --- | --- | --- |
| Amino acid | 66.67 | 83.03 | 81.67 | 0.34 |
| Anion | 50.00 | 74.41 | 72.78 | 0.14 |
| Cation | 75.00 | 72.92 | 73.33 | 0.40 |
| Electron | 90.00 | 77.65 | 78.33 | 0.35 |
| Protein/mRNA | 93.33 | 80.61 | 81.67 | 0.47 |
| Sugar | 83.33 | 74.40 | 75.00 | 0.32 |
| Other | 55.00 | 65.00 | 63.89 | 0.13 |
| Non-transporter | 73.33 | 82.50 | 79.44 | 0.55 |
