# Supplementary material for: Prediction of Membrane Transport Proteins and Their Substrate Specificities Using Primary Sequence Information
Source: PLoS One. 2014 Jun 26;9(6):e100278. doi: 10.1371/journal.pone.0100278 (PMC4072671; doi:10.1371/journal.pone.0100278)
Supplement: Table S5 — The performances of the hybrid AAIndex and SwissProt-based PSSM models on the independent dataset. (DOCX) [file pone.0100278.s006.docx]

**Table S5**. The performances of the hybrid AAIndex and SwissProt-based PSSM models on the independent dataset.

| **Transporter class** | **Sensitivity** | **Specificity** | **Accuracy** | **MCC** |
| --- | --- | --- | --- | --- |
| Amino acid | 80.00 | 84.85 | 84.44 | 0.44 |
| Anion | 66.67 | 68.45 | 68.33 | 0.19 |
| Cation | 77.78 | 69.44 | 71.11 | 0.39 |
| Electron | 80.00 | 81.76 | 81.67 | 0.34 |
| Protein/mRNA | 93.33 | 82.42 | 83.33 | 0.49 |
| Sugar | 83.33 | 80.36 | 80.56 | 0.37 |
| Other | 65.00 | 70.00 | 69.44 | 0.23 |
| Non-transporter | 75.00 | 82.50 | 80.00 | 0.56 |
