# Supplementary material for: Prediction of Membrane Transport Proteins and Their Substrate Specificities Using Primary Sequence Information
Source: PLoS One. 2014 Jun 26;9(6):e100278. doi: 10.1371/journal.pone.0100278 (PMC4072671; doi:10.1371/journal.pone.0100278)
Supplement: Table S6 — Confusion matrix of best SVM models (AAindex+PSSM) on main dataset. (DOCX) [file pone.0100278.s007.docx]

**Table S6.** Confusion matrix of best SVM models (AAindex + PSSM) on main dataset.

|  | Amino | Anion | Cation | Electron | Protein | Sugar | Other | Non-transporter |
| --- | --- | --- | --- | --- | --- | --- | --- | --- |
| Amino | 60 | 1 | 1 | 0 | 2 | 1 | 3 | 2 |
| Anion | 3 | 43 | 1 | 3 | 2 | 0 | 5 | 3 |
| Cation | 11 | 8 | 182 | 6 | 6 | 7 | 21 | 19 |
| Electron | 1 | 2 | 0 | 51 | 0 | 1 | 3 | 2 |
| Protein | 3 | 1 | 2 | 2 | 52 | 2 | 5 | 3 |
| Sugar | 1 | 2 | 1 | 0 | 2 | 46 | 5 | 3 |
| Other | 9 | 11 | 9 | 6 | 9 | 10 | 123 | 23 |
| Non-transporter | 15 | 21 | 18 | 13 | 17 | 15 | 23 | 478 |
