# Supplementary material for: Prediction of Membrane Transport Proteins and Their Substrate Specificities Using Primary Sequence Information
Source: PLoS One. 2014 Jun 26;9(6):e100278. doi: 10.1371/journal.pone.0100278 (PMC4072671; doi:10.1371/journal.pone.0100278)
Supplement: Table S7 — Confusion matrix of best SVM models (AAindex+PSSM) on independent dataset. (DOCX) [file pone.0100278.s008.docx]

**Table S7.** Confusion matrix of best SVM models (AAindex + PSSM) on independent dataset.

|  | Amino | Anion | Cation | Electron | Protein | Sugar | Other | Non-transporter |
| --- | --- | --- | --- | --- | --- | --- | --- | --- |
| Amino | 14 | 0 | 0 | 0 | 0 | 0 | 1 | 0 |
| Anion | 0 | 9 | 0 | 0 | 1 | 0 | 1 | 1 |
| Cation | 1 | 1 | 27 | 1 | 2 | 0 | 3 | 1 |
| Electron | 0 | 1 | 0 | 8 | 0 | 0 | 1 | 0 |
| Protein | 0 | 0 | 0 | 0 | 14 | 0 | 1 | 0 |
| Sugar | 0 | 0 | 0 | 0 | 1 | 11 | 0 | 0 |
| Other | 0 | 2 | 2 | 1 | 0 | 1 | 12 | 2 |
| Non-transporter | 1 | 3 | 3 | 2 | 0 | 0 | 5 | 46 |
